# Plant trait and vegetation data along a 1314 m elevation gradient with fire history in Puna grasslands, Perú

**DOI:** 10.1038/s41597-024-02980-3

**Published:** 2024-02-21

**Authors:** Aud H. Halbritter, Vigdis Vandvik, Sehoya H. Cotner, William Farfan-Rios, Brian S. Maitner, Sean T. Michaletz, Imma Oliveras Menor, Richard J. Telford, Adam Ccahuana, Rudi Cruz, Jhonatan Sallo-Bravo, Paul Efren Santos-Andrade, Lucely L. Vilca-Bustamante, Matiss Castorena, Julia Chacón-Labella, Casper Tai Christiansen, Sandra M. Duran, Dagmar D. Egelkraut, Ragnhild Gya, Siri Vatsø Haugum, Lorah Seltzer, Miles R. Silman, Tanya Strydom, Marcus P. Spiegel, Agustina Barros, Kristine Birkeli, Mickey Boakye, Fernanda Chiappero, Adam Chmurzynski, Josef C. Garen, Joseph Gaudard, Tasha-Leigh J. Gauthier, Sonya R. Geange, Fiorella N. Gonzales, Jonathan J. Henn, Kristýna Hošková, Anders Isaksen, Laura H. Jessup, Will Johnson, Erik Kusch, Kai Lepley, Mackenzie Lift, Trace E. Martyn, Miguel Muñoz Mazon, Sara L. Middleton, Natalia L. Quinteros Casaverde, Jocelyn Navarro, Verónica Zepeda, Korina Ocampo-Zuleta, Andrea Carmeli Palomino-Cardenas, Samuel Pastor Ploskonka, Maria Elisa Pierfederici, Verónica Pinelli, Jess Rickenback, Ruben E. Roos, Hilde Stokland Rui, Eugenia Sanchez Diaz, Andrea Sánchez-Tapia, Alyssa Smith, Erickson Urquiaga-Flores, Jonathan von Oppen, Brian J. Enquist

**Affiliations:** 1 Department of Biological Sciences, University of Bergen, Bergen, Norway; 2 Bjerknes Centre for Climate Research, University of Bergen, Bergen, Norway; 3 Department of Biology and Sabin Center for Environment and Sustainability, Wake Forest University, Winston-Salem, NC USA; 4 Department of Ecology and Evolutionary Biology, University of Arizona, Tucson, AZ USA; 5 Department of Botany and Biodiversity Research Centre, The University of British Columbia, Vancouver, Canada; 6 AMAP, Université de Montpellier, Montpellier, France; 7 School of Geography and the Environment, University of Oxford, Oxford, United Kingdom; 8 Universidad Nacional de San Antonio Abad del Cusco, Cusco, Perú; 9 Department of Biology, University of Copenhagen, Copenhagen, Denmark; 10 Department of Forest and Rangeland Stewardship, Fort Collins, CO USA; 11 Department of Forest and Rangeland Stewardship, Colorado State University, Fort Collins, CO USA; 12 Département de sciences biologiques, Université de Montréal, Montréal, Canada; 13 Instituto Argentino de Nivología y Glaciología y Ciencias Ambientales, CONICET y Universidad Nacional de Cuyo, Mendoza, Argentina; 14 School of Geography, Planning, and Spatial Sciences, University of Tasmania, Hobart Tasmania, Australia; 15 Department of Environmental Science Policy and Management, University of California, Berkeley, CA USA; 16 Department of Geography & Environmental Management, University of Waterloo, Waterloo Ontario, Canada; 17 Departamento de Ecología, Facultad de Ciencias Biológicas, Pontificia Universidad Católica de Chile, Santiago, Chile; 18 Institute of Arctic and Alpine Research, University of Colorado Boulder, Boulder, CO USA; 19 Department of Biological and Environmental Sciences, University of Gothenburg, Gothenburg, Sweden; 20 Department of Botany, Charles University in Prague, Praha, Czech Republic; 21 Department of Biosciences, University of Oslo, Oslo, Norway; 22 Department of Forestry and Natural Resources, Purdue University, West Lafayette, IN USA; 23 Newcastle University, Newcastle, United Kingdom; 24 School of Geography, Development & Environment, University of Arizona, Tucson, AZ USA; 25 School of Biological Sciences, University of Queensland, Queensland, Australia; 26 Faculty of Environmental Sciences and Natural Resource Management, Norwegian University of Life Sciences, Ås, Norway; 27 Department of Biology, University of Oxford, Oxford, United Kingdom; 28 Science Division, New York Botanical Garden, Bronx, NY USA; 29 Departamento de Ecología y Recursos Naturales, Universidad Nacional Autónoma de México, Mexico City, Mexico; 30 Programa de Doctorado en Ciencias mención Ecología y Evolución, Universidad Austral de Chile, Santiago, Chile; 31 Department of Earth and Environmental Sciences, Katholieke Universiteit Leuven, Leuven, Belgium; 32 Departamento de Ecología y Gestión Ambiental, Universidad de la República, Maldonado, Uruguay; 33 School of Geosciences, University of Edinburgh, Edinburgh, Scotland; 34 Tropical Diversity, Royal Botanic Garden Edinburgh, Edinburgh, UK; 35 Norwegian Institute for Nature Research, Oslo, Norway; 36 Instituto de Pesquisas Jardim Botânico do Rio de Janeiro, Rio de Janeiro, Brazil; 37 Department of Systematic and Evolutionary Botany, University of Zurich, Zurich, Switzerland; 38 Department of Biology, Aarhus University, Aarhus, Denmark; 39 Present Address: Pontificia Universidad Católica del Peru, Lima, Perú

**Keywords:** Ecology, Plant sciences, Ecophysiology, Grassland ecology, Ecosystem ecology

## Abstract

Alpine grassland vegetation supports globally important biodiversity and ecosystems that are increasingly threatened by climate warming and other environmental changes. Trait-based approaches can support understanding of vegetation responses to global change drivers and consequences for ecosystem functioning. In six sites along a 1314 m elevational gradient in Puna grasslands in the Peruvian Andes, we collected datasets on vascular plant composition, plant functional traits, biomass, ecosystem fluxes, and climate data over three years. The data were collected in the wet and dry season and from plots with different fire histories. We selected traits associated with plant resource use, growth, and life history strategies (leaf area, leaf dry/wet mass, leaf thickness, specific leaf area, leaf dry matter content, leaf C, N, P content, C and N isotopes). The trait dataset contains 3,665 plant records from 145 taxa, 54,036 trait measurements (increasing the trait data coverage of the regional flora by 420%) covering 14 traits and 121 plant taxa (ca. 40% of which have no previous publicly available trait data) across 33 families.

## Background & Summary

Mountains cover 27% of the world’s land surface, and they play a key role in harbouring and maintaining global biodiversity and in delivering indispensable ecosystem functions and benefits to people^1–4^. High-elevation mountain regions around the world support characteristic alpine ecosystems^5,6^, and as these ecosystems are temperature-limited they are susceptible to anthropogenic climate change, especially as high-elevation climates are warming faster than global averages^7^. As a result, alpine ecosystems and biodiversity are particularly threatened by climate change, as evidenced by ongoing shifts in species distributions and phenology, ecological communities, and carbon, nutrient, and water cycling^8,9^. Knowledge of the distributions and functioning of alpine biota and ecosystems is crucial to predict and mitigate future global change impacts on mountain ecosystems, as well as for the human societies that depend on these systems for livelihoods and other ecosystem functions and services^2,3^.

Functional traits can improve our mechanistic understanding of species’ responses to and functioning under environmental change by linking individuals’ phenotypes and the environment^10,11^. Thus, trait-based approaches can provide insights into how species and communities respond to climate changes and how community changes, in turn, impact ecosystem functioning^12–14^. For example, traits can inform process-based understanding of the impacts of global climate changes on biodiversity and they can elucidate feedback mechanisms between ecosystems and global carbon, nutrient, and water cycles^15^. Explicitly quantifying intraspecific trait variation can provide valuable insights into ecological and evolutionary processes - including community and population responses, plasticity, and local adaptations - that underpin observed community patterns and global change impacts^16–18^. Traits associated with plant size^19^ and the leaf economics spectrum (a set of intercorrelated traits that characterise species along an axis ‘fast’ to ‘slow’ photosynthetic and tissue turnover rates and life histories)^20–22^, should be particularly relevant for responses to climatic warming.

The alpine Puna and Paramo grasslands of the high Andes, which cover an area of 470,000 km^2^, are a global biodiversity hotspot and provide globally and regionally important supporting and regulating ecosystem services such as water supply and carbon (C) sequestration^23–26^. Due to a continuous growing season and frequent water-logging, humid tropical alpine grassland ecosystems such as the Puna are globally important carbon stores that accumulate more than 250 Mg ha^−1^ of C^27,28^. People have used the Puna and Paramo grasslands for provisioning and cultural services, including hunting, grazing by domesticated ungulates, transport, and crop production, since pre-Inca times^29^. These ecosystems and their ecosystem functions and benefits to people are now threatened by climate change in combination with increasing human pressures associated with land-use change and other global change drivers^23,25,30^.

While climate change projections are uncertain for high-altitude regions of the Andes, warming and associated increased risk of hot extremes are expected to continue^7^, with potential to cause system-wide changes in alpine ecosystems throughout the Andes, including the Puna and Paramo grasslands^25,26,31^. Specifically, advancing treelines are likely to reduce the area of the alpine vegetation, including grasslands, increasing the risk of biodiversity loss and extinctions of endemic taxa^23^. Changes in precipitation patterns and increased evapotranspiration will likely increase soil carbon turnover and decrease below-ground organic carbon storage impacting the water supply^23^. Agriculture and livestock may expand into higher elevations, potentially increasing fire frequencies due to burning to improve forage for livestock^32,33^. However, there is limited empirical data on the combined impact of climate and land-use change on the Puna and Paramo grasslands and their biodiversity and functioning^26^.

**Fig. 1:**
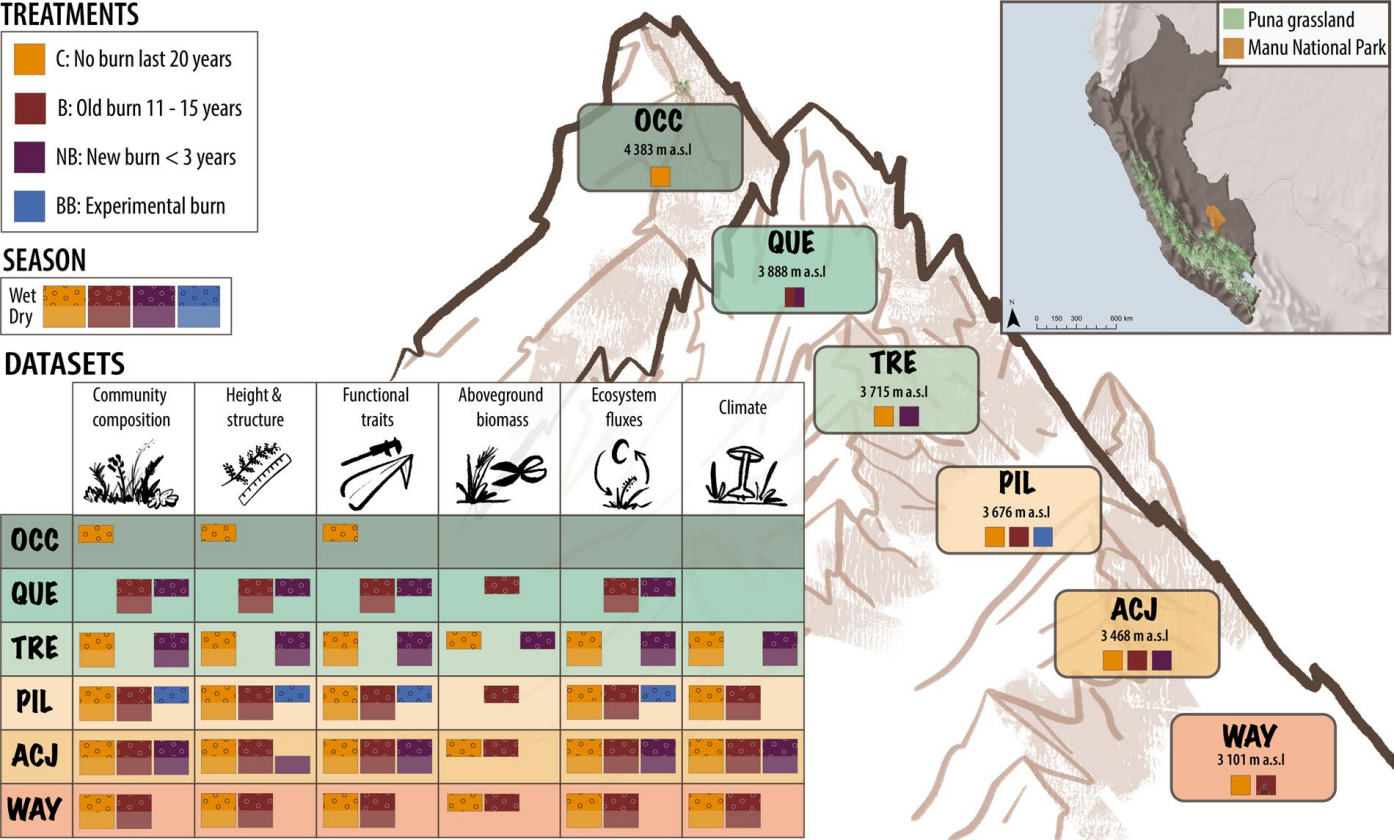
Study sites and fire treatments along the elevational gradient in the Puna grasslands of the southeastern Andes, in the Manú National Park buffer zone, Department of Cusco, Paucartambo province, Challabamba district, Perú. The inset table shows the datasets available for each site (green to reddish boxes), treatment (yellow, brown, and blue squares within sites), and season (dark dropped vs. lighter faded rectangles within squares). Note that at QUE there is only one box because the site burnt in November 2019 and the plots thus changed from burnt to recently burnt. Datasets are further described in Table 1 and Fig. 2. The inset map on the top right shows the location of Manú National Park in Perú.

This paper reports a comprehensive plant functional trait dataset collected from Puna grasslands with different fire histories along a 1314 m elevational gradient from 3072 to 4386 m above sea level (a.s.l.) in Perú. Across six study sites and 12 unique elevation x fire history treatments (Fig. 1), we collected data on structural, leaf economic, and chemical plant functional traits and associated plant community composition, species richness, vegetation cover, height and biomass, ecosystem fluxes, and microclimate in all sites and treatments and during the wet and dry seasons between 2018 and 2020 (Table 1). These data provide a baseline for understanding how variation in elevation and fire history affect plant traits and ecosystem dynamics in the Puna grasslands, an ecosystem crucial for biodiversity and ecosystem services across the Andes. This research can serve as a foundation for future research to monitor changes and inform conservation strategies, particularly in the face of climate change and human-induced disturbances in these sensitive ecosystems. Additionally, we hope that these rich datasets from an undersampled region will be valuable for global comparisons of alpine vegetation.

**Table 1:** Description and location of the datasets on Puna grassland plant functional traits and associated data from an elevational gradient in the Manú National Park buffer zone, Department of Cusco, Paucartambo province, Challabamba district, Perú.

Dataset	Response variable	Number of data points^a^ and taxa^b^	Temporal range	Citation for raw data, clean data and code
i	Plant community composition	3,665^a^ 145^b^	2018–2020	Raw data^70^, clean data^70^, code^71^
ii	Vegetation height and structure	1,627^a^	2018–2020	Raw data^70^, clean data^70^, code^71^
iii	Plant functional traits	54,036^a*^ 121^b^	2018–2020	Raw data^70^, clean data^70^, code^71^
iv	Aboveground biomass	129^a^	2019	Raw data^70^, clean data^70^, code^71^
v	Ecosystem fluxes	Ecosystem CO_2_ flux: 609^a^ Soil respiration:455^a^ Evapotranspiration: 609^a^	2018–2020	Raw data^70^, clean data^70^
vi	Climate	761,624^a^	2019–2020	Raw data^70^, clean data^70^, code^71^

This table summarises information on dataset number, response variable(s), number of observations, the data’s temporal range, location of the primary data, the final published data, and the code for extracting and cleaning data from the primary data.

*Note that the number of trait observations will increase when last samples are processed in the lab. Due to Covid-19, the leaves from the last data collection campaign have been stuck in Perú.

We collected plant functional traits for 73.8% of the species encountered in the Puna grassland plant communities across our study sites, including data on intraspecific trait variation for the dominant species. The resulting dataset (Table 1) encompasses 54,036 trait measurements from 121 taxa, which extends existing trait data from the regional flora by ca. 36 additional species and increases the number of unique trait measurements from this regional flora by 420%, relative to the public TRY database^34^. Our data were collected as part of two international Plant Functional Traits Courses^35^ (PFTC3 and PFTC5) for international students in trait-based theory and methods^36,37^ with additional campaigns (PUNA) to augment the data across years and seasons. The data are comparable with data from PFTC courses in China^38^, Svalbard^39^, and Norway and with data from upcoming courses (see https://plantfunctionaltraitscourses.w.uib.no/), providing a resource for integrated regional assessment of traits, community assembly, and ecosystem functioning, and for future cross-regional comparative studies.

## Methods

### Data management and workflows

**Fig. 2:**
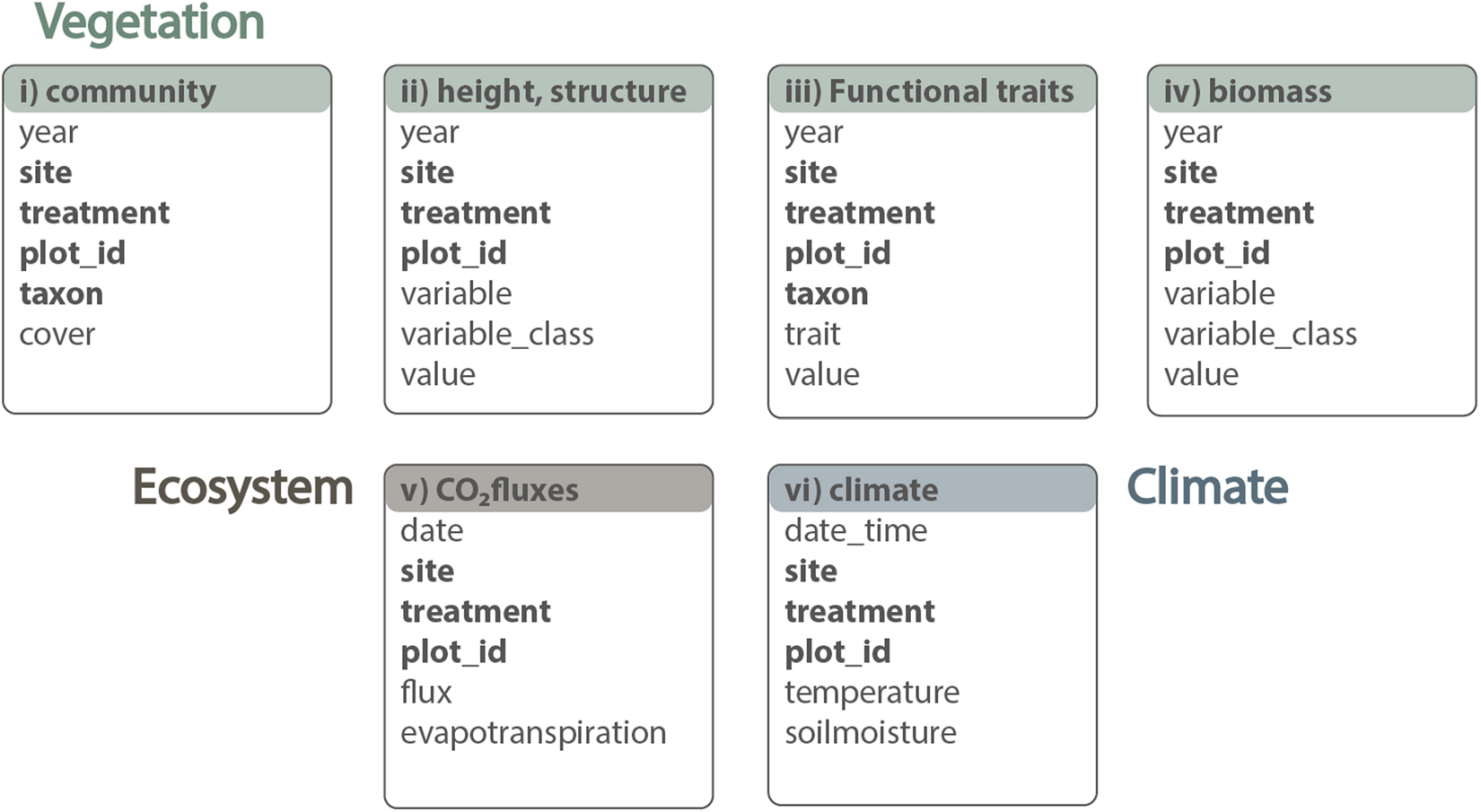
Data structure from the elevational gradient and fire treatment study in the Puna grasslands of the southeastern Andes, Manú National Park buffer zone, Department of Cusco, Paucartambo province, Challabamba district, Perú. The boxes represent data tables including community composition (dataset i), community height and structure (dataset ii), plant functional traits (dataset iii), biomass (dataset iv), ecosystem fluxes (dataset v), and climate (dataset vi). Names of individual data tables are given in the title area, and a selection of the main variables available within tables are given in the internal lists. For complete sets of variables for each dataset, see Tables 2–7. Note that all bold variables are shared between several tables and can be used as keys to join them.

Our approach to research planning, execution, reporting, and management follows best-practice approaches to open and reproducible science, as described and advocated in e.g.^40–43^. Specifically, we use community-approved standards for experimental design and data collection, we clean and manage the data using a fully scripted and reproducible data workflow, and we deposit data and code in open repositories. For details, see Fig. 2 in^44^. Our Puna grassland data consists of six main data tables linked by keys related to time, sampling locations, treatments, species, replicate plots and individuals (Fig. 2).

### Research site selection and basic site information

The study was conducted in the Puna grasslands of the Peruvian southeastern Andes, in the Manú National Park buffer zone, Department of Cusco, Paucartambo province, Challabamba district, Perú. The Puna grasslands are located above the upper treeline limit of the cloud forest. At the border between the cloud forest and the Puna grassland (c. 3000 m a.s.l.), the annual rainfall is approximately 1560 mm, and the mean annual air temperature is 11.8 °C^45^. The dry season in these systems is between May/June and August/September, and although there is little rain, fog from the rainforest provides ample moisture. The Puna grasslands are dominated by tussock-forming grasses, the dominant genera being *Calamagrostis, Stipa*, and *Festuca*^46^. The Puna is a cultural landscape traditionally used for free-range livestock grazing. While there is no grazing inside the Manu National Park, the surrounding local communities commonly use the buffer area for grazing cattle, and sometimes livestock does enter and graze within the park^28^. The soils have deep organic layers^27,47^ (20 cm on average, but they can be as much as 110 cm deep, Oliveras pers. obs.).

We selected six sites along an elevational gradient above the cloud forest treeline (Fig. 1), and in March 2019, we established sites at Wayqecha (WAY; 3101 m a.s.l.), Acjanaco (ACJ; 3468 m a.s.l.), Pilco Grande (PIL; 3676 m a.s.l.), Tres Cruzes (TRE 3715 m a.s.l.) and Quello Casa (QUE; 3888 m a.s.l.). Latitude and longitude were recorded for each site. WAY belongs to the Private Conservation Area Wayqecha managed by ACCA^46^, while all the other sites are located within the protected area of Manu National Park. In April 2019, we established a sixth site, Ocoruro (OCC; 4383 m a.s.l.), located in the Calca province, outside Manu National Park.

### Fire treatments

At each site, we selected areas that differed in the time since the last burning: No burning in the last 20 years (C; control), 11–15 years since burning (B; old burn), and <3 years since burning (NB; new burn), and experimental burn (BB), see^46,48,49^ (Fig. 1). All sites except QUE had control plots, all sites except TRE and OCC had old burnt treatment, all sites except OCC, QUE and WAY had newly burnt areas, and we also sampled an area at PIL that was experimentally burnt in 2006 and than again in 2013 (BB; experimental burn). Note that the QUE site burnt in November 2019 and thus changed from a burnt to a recently burnt site.

### Plot selection and data collection

We installed five 1.2 m × 1.2 m plots within each burning treatment at each site (i.e., n = 5–15 per site). At PIL, only three plots were installed in the experimentally burnt area (BB) due to space limitations. We marked the corners of each plot permanently with metal sticks. Data were collected between March 2018 and March 2020, during the two plant functional traits courses (referred to by their course numbers; PFTC3, PFTC5) with multiple additional data collection campaigns (referred to as the PUNA project) to allow data collection during the wet season (March 2018 and 2020, April 2019) and dry season (July and November 2019). The total number of plots is 63.

### Species identification, taxonomy, and flora

All species sampled for vegetation and functional traits were identified in the field. Plants or vouchers were collected for identification checks using the literature^50–52^, and specimens that were difficult to identify were brought back to the Cusco University for identification and deposition of vouchers by one of the co-authors (LLVB). Some species were only identified to genus or family level due to difficulties with identifying sterile graminoids or young plants due to recent burn events. All taxon names were standardised using the TNRS R package^53^ based on the Taxonomic Name Resolution Service^54^, Tropicos^55^, The Plant List^56^, and USDA^57^ databases.

## Dataset (i): Plant community composition sampling

All vascular plant species in each plot were surveyed in March 2018 and re-surveyed in April, July, and November 2019. As some recently burnt sites were installed in 2019 (ACJ, TRE, QUE), they had fewer surveys and were additionally surveyed in March 2020. We used a 1.2 m × 1.2 m frame overlain with a grid of 25 subplots. During each survey, we estimated the percentage coverage of each species in the plot to the nearest 1%, and we also recorded if the species present were fertile (i.e., contained buds, flowers, seeds) and the occurrence of seedlings. Note that the total coverage in each plot can exceed 100 due to the layering of the vegetation. Identifications were checked with available literature and by experts (see description above and the Technical Validation and Usage notes below for details).

## Dataset (ii): Vegetation height and structure sampling

Vegetation structure data for each of the 63 vegetation plots were recorded at each plant community composition campaign (see above). Minimum, median, and maximum vegetation height and bryophyte depth were measured using a ruler at five evenly spaced points per plot. We also recorded the total percent coverage of graminoids, forbs, shrubs, bryophytes, lichens, litter, bare ground, and bare rocks.

## Dataset (iii): Plant functional traits sampling and lab analyses

### Plot-level sampling for leaf trait analyses

We collected whole plants for leaf trait analyses from all treatments within each site in multiple campaigns in March 2018 and April 2019, during the wet season, and July and November 2019, during the dry season, except for the OCC site, where plants were only collected once in April 2019. At each campaign, we sampled traits from up to five individuals of all species present in each plot, if possible. Sampling was done outside the experimental plots, within a transect 50 m to each side of the plot. To avoid repeated sampling from a single clone, we selected individuals visibly separated from other ramets of that species. In line with community standards^58^, the consecutive trait campaigns aimed to obtain trait data from species cumulatively making up at least 80% of the vegetation cover, and as we were interested in intraspecific trait variation, we aimed to achieve this with local trait measurements in both control and burnt plots at each site during both the dry and wet seasons. In March 2020, we collected additional traits from the sites as needed, focusing on the recently burnt plots at ACJ, TRE, QUE, and associated control plots at ACJ and TRE because they were installed later and thus contained fewer trait data (see above).

### Intraspecific trait variability sampling for leaf trait analyses

To further explore intraspecific and intraindividual trait variability, we collected leaves from several individuals of selected species at three sites along the elevation gradient in the control treatments in 2020 (WAY, AJC and TRE). For this we selected six species (*Halenia umbellata, Lachemilla orbiculata*, *Paspalum bonplandianum, Rhynchospora macrochaeta*, *Gaultheria glomerata and Vaccinium floribundum*) that were abundant along the whole gradient. At each site, two individuals were randomly chosen in a band spanning 5–10 m to the left and right of each plot, resulting in 10 individuals per species per site. When two individuals could not be sampled at each plot, more were sampled from other plots in the same site, aiming for 10 individuals per site, but fewer when this was not possible. All individuals of the same species were at least two metres apart to ensure the same genetic individual was not sampled multiple times.

### Processing and storage

The sampled plant individuals were labeled, put in plastic bags with moist paper towels, and stored in darkness at 4 °C until further processing. Processing was generally done the day after plant collection in the field, but some specimens were stored for up to 4 days. Before processing, plant identification was checked (see above). Up to three healthy, fully expanded leaves were sampled from each individual. The leaves were cut off as close to the stem as possible, including the blade, petiole, and stipules when present. For *Lycopodiella*, *Lycopodium*, and *Hypericum* species, which have thin and needle-shaped leaves, and for *Baccharis* species, which have wing-shaped leaves attached to the stem, an 8–11 cm stem section was cut off, including side shoots where present, and all leaves from this section were removed and used one sample. For *Vaccinium floribundum*, which has tiny leaves, we sampled 5–10 leaves per sample. Further processing was completed within 24 hours (see below).

### Plant functional trait measurements

We measured 14 leaf functional traits that are related to potential physiological growth rates and environmental tolerance of plants, following the standardised protocols in Pérez-Harguindeguy *et al*.^58^: plant height (cm), leaf wet mass (g), leaf dry mass (g), leaf area (cm^2^), leaf thickness (mm), leaf dry matter content (LDMC, g/g), specific leaf area (SLA, cm^2^/g), carbon (C, %), nitrogen (N, %), phosphorus (P, %), carbon-nitrogen ratio (C:N), nitrogen-phosphorus ratio (N:P), carbon isotope ratio (δ^13^C, ‰), and nitrogen isotope ratio (δ^15^N, ‰). Initial leaf processing was done at the Wayqecha Biological Station in the Paucartambo Province, Cusco Region, Perú. Processing was done in the following steps:

1. **Plant height**. Before collecting the leaves in the field, standing height (measured in cm) was measured for each individual from the ground to the tallest vegetative organ without stretching. For graminoids we measured both standing height and stretched height, which is equivalent to leaf length (the stretched height was measured in the field or the lab during processing).
2. **Leaf wet mass**. Each leaf (including blade, petiole, and stipules when present) was weighed to the nearest 0.001 g to assess fresh mass.
3. **Leaf area**. Leaves (including blade, petiole, and stipules when present) were carefully patted dry with paper towels, flattened (folded to their maximum area), and scanned using a Canon LiDE 220 flatbed scanner at 300dpi. Leaves that grow naturally folded (e.g., some *Agrostis, Calamagrostis, Carex, Festuca*, and *Trichophorum* species) were scanned as such, thereafter, the area was multiplied by two during data processing. Any dark edges on the scans were automatically cropped during data processing. Leaf area was calculated using ImageJ^59^ and the LeafArea package^60^.
4. **Leaf thickness**. Leaf thickness was measured at three locations on each leaf blade with a digital caliper (Micromar 40 EWR, Mahr) and averaged for further analysis. When possible, the three measurements were taken on the middle vein of the leaf and lamina with and without veins. The petiole or stipule thickness was not measured.
5. **Leaf dry mass**. Leaves (including blade, petiole, and stipules when present) were dried for at least 72 hours at 65 °C before dry mass was measured to the nearest 0.0001 g.
6. We calculated **specific leaf area** (SLA) by dividing leaf area by dry mass and **leaf dry matter content** (LDMC) as the ratio of leaf dry and wet mass.
7. **Leaf stoichiometry and isotopes**. Leaf stoichiometry and isotope assays (P, N, C, δ^15^N, and δ^13^C) were conducted for a subset of the leaves. These leaves were stored in a drying oven at 65 °C and then transported to the University of Arizona for analyses. First, each leaf (including blade, petiole, and stipules when present) was ground into a fine homogenous powder. Total phosphorus concentration was determined using persulfate oxidation followed by the acidmolybdate technique (APHA 1992), and phosphorus concentration was then measured colorimetrically with a spectrophotometer (TermoScientifc Genesys20, USA). Nitrogen, carbon, stable nitrogen (δ^15^N), and carbon (δ^13^C) isotopes were measured at the Department of Geosciences Environmental Isotope Laboratory at the University of Arizona. Samples of 1.0 ± 0.2 mg were combusted in a Costech elemental analyser and measurements were made on a continuous-flow gas-ratio mass spectrometer (Finnigan Delta PlusXL). Standardisation was based on acetanilide for elemental concentration, NBS-22 and USGS-24 for δ^13^C, and IAEA-N-1 and IAEA-N-2 for δ^15^N. Precision is at least ± 0.2 for δ^15^N (1 s), based on repeated internal standards. In addition to measurements, ratios between C:N and N:P are also reported. At the time of publication, 754 leaves have been processed for chemical traits. More leaves are available and will be added to the dataset as processed.

## Dataset (iv): Above-ground biomass

Biomass data were collected in April 2019 from extra plots set up in the control and burnt area in WAY and ACJ, the control and newly burnt area in TRE, the burnt area in PIL and QUE, for a total of eight plots. At each site and treatment, biomass was harvested from one 1.2 m × 1.2 m plot close to the existing vegetation plots for a total of eight plots. For each plot, vegetation height and structure were sampled as described above (see dataset ii). All aboveground vegetation in the plot was then cut 2–5 cm above the ground and sorted into functional groups (graminoids, forbs, woody, fern, moss and bryophytes). The biomass was dried at 60 °C for 48 hours and weighed.

## Dataset (v): Ecosystem fluxes (CO_2_ and H_2_O)

### Plot-level flux measurements

We used a closed-system tent setup to measure ecosystem CO_2_ to assess net ecosystem exchange (NEE), ecosystem respiration (R_eco_), and gross primary productivity (GPP) and ecosystem H_2_O fluxes to estimate evapotranspiration (ET) and evaporation (E). Each flux measurement consists of a paired light/dark measurement from which we calculated the ecosystem fluxes following Sloat *et al*.^61^. Briefly, CO_2_ fluxes under light conditions measure NEE, including photosynthesis and R_eco_ (including both plant and soil respiration), whereas fluxes under dark conditions measure Reco only (again, both plant and soil). As NEE = GPP - R_eco_, these measurements can be used to calculate GPP^62^. Similarly, the increase in water vapour in the tent during measurements is due to both evaporation (E) and transpiration (T) of water. Note that E within the tent reflects evaporation of water to the air from sources such as the soil, canopy, and any water surfaces within the chamber, whereas T reflects water movement within plants and the subsequent water loss as vapour through stomata. Thus, H_2_O fluxes under light conditions reflect ET, whereas measurements under dark conditions represent E only, and as ET = E + T, these rates can be used to calculate T^62^.

The closed-system setup used for these measurements was constructed as a cuboid PVC frame (plot footprint 1.2 m × 1.2 m; volume 2.197 m^3^) which was covered with a tight-fitting tent made of translucent ripstop polyethylene fabric that transmits ~75% of photosynthetically active radiation (PAR) while limiting heat buildup (Shelter Systems, see^63–65^). The tent had a ca. 30 cm wide skirt around the edge that was weighed down with a heavy chain to seal the cuboid during measurements. For dark measurements, the tent was covered with a light-impermeable black tarp. Ecosystem CO_2_ and H_2_O fluxes were measured with a Li-Cor 7500 CO_2_/H_2_O infrared gas analyzer (IRGA) mounted on a tripod (LI-COR Inc., Lincoln, NE, USA) with two DC-powered fans used to mix the air within the chamber.

Each paired light/dark flux measurement was conducted in the following steps: We (i) placed the IRGA and fans within the plot (ii) measured ambient CO_2_ and H_2_O for 90 s, (iii) placed the tent over the plot and sealed it against the soil surface (iv) measured CO_2_ and H_2_O within the tent under light conditions for 90 s, (iv) removed the tent from the plot for 2 minutes to allow both the tent and the vegetation to equilibrate with the outside air, (v) placed the tent on the plot and covered it with the light-impenetrable tarp within 30-seconds (vi) measured CO_2_ and H_2_O within the tent under dark conditions for 90 s. Previous studies have shown that the pressure gradient caused by changing concentrations of CO_2_ and H_2_O in the closed system begins to affect stomatal conductance after about 90 s^63^. Measuring for this relatively short time also mitigates the effect of increasing temperature on the plants under the tent.

We measured CO_2_ and H_2_O fluxes once in each plot/treatment/site combination during each campaign in March 2018, April 2019, July 2019, November 2019, and March 2020. All plot flux measurements were done during the peak photosynthetic period of the day. As not all measurements could be made under full sun conditions, light response curves are also provided. Light-response data are available for GPP standardisation for some plots, sites, and treatments in April 2019, July 2019, November 2019, and March 2020. Each light-response curve consists of one measurement in full light, three at different levels of shading using layers of white tulle, and one in full darkness using the black tarp described above^66^.

### Soil respiration measurements

For measuring soil CO_2_ fluxes, i.e., soil respiration (Rs), we used an LI-840 infrared gas analyzer (LI-COR Inc., Lincoln, NE, USA), connected to custom soil respiration chambers made of PVC tubing (hereafter PVC collars), installed approximately three weeks before the first measurement in 2018. We inserted two PVC collars into the ground in all plots. Each PVC collar was approximately 8–10 cm in diameter and created a soil chamber ~1 L headspace volume. To adjust for topographic heterogeneity, we measured the height of each collar at four points, using the mean height to calculate the exact volume of the collar. Each soil collar was securely fitted with a custom polyethylene lid to ensure a closed chamber. The lid has the same diameter as the collars minus a couple of mm to allow for a proper seal. The concentration of CO_2_ within the soil respiration chambers was recorded for approximately 90 seconds. These measurements were done during the same campaigns as for CO_2_ and H_2_O fluxes.

### Environmental measurements

For each flux measurement, we measured environmental data. We measured photosynthetic active radiation (PAR; µmol photons m-2 s-1) within the tent approximately every 15 seconds during the 90-second measuring interval using a quantum sensor (Li-190, LI-COR Biosciences, Lincoln, NE, USA). Soil moisture (% volume) was measured at five points evenly distributed within each plot and twice adjacent to each soil respiration collar just after each flux measurement. Soil temperature (°C) was measured using a digital thermometer with an accuracy of ±0.1 °C at two locations within each plot and each soil respiration collar during all CO_2_ flux measurements. Vegetation canopy temperature (°C) was measured for each ecosystem flux measurement with an IR thermometer with a laser pointer. Five measurements were made evenly distributed across the plot just after each flux measurement for each plot.

### Calculations

All measurements were visually evaluated for quality, and only measurements that showed a consistent linear relationship between CO_2_ and time for at least 60 s were used for NEE calculations. NEE was calculated using a linear model from the temporal change of CO_2_ concentration within the closed chamber following Jasoni *et al*.^67^, using this equation:

1 $$NEE=\frac{\delta CO2}{\delta t}\times \frac{P\times V}{R\times A\times (T+273.15)}$$

where *δ*CO_2_/*δt* is the slope of the CO_2_ concentration against time (µmol mol-1 s-1), P is the atmospheric pressure (kPa), R is the gas constant (8.314 kPa m3 K-1 mol-1), T is the air temperature inside the chamber (°C), V is the chamber volume (m^3^), A is the surface area (m^2^). We also used a non-linear approach for the same calculation based on the “leaky chamber” method developed by Saleska *et al*.^68^. Note that we define NEE such that negative values reflect CO_2_ release from the ecosystem to the atmosphere, whereas positive values reflect CO_2_ uptake in the ecosystem, and that we provide the measured fluxes only, as GPP and T can be calculated from these fluxes.

## Dataset (vi): Climate data

Climate data including air temperature (15 cm), ground temperature (0 cm), soil temperature (−5cm), and soil moisture (−5cm) were recorded using TOMST TMS-4 data loggers^69^. Climate data were measured between April 2019 and March 2020 in 2–4 plots per site and treatment (see Fig. 1), except for OCC, QUE, and the BB treatment. The raw soil moisture data were converted to soil moisture using an intermediate soil type, “sandy loam A” provided by TOMST. The raw soil moisture values can be accessed, note that other conversions are possible.

## Additional Data

We also measured photosynthesis-light response curves for *Paspalum bonplandianum* and *Gaultheria glomerata*, and photosynthesis-temperature response curves for *Paspalum bonplandianum*, *Rhynchospora macrochaet*a, and *Gaultheria glomerata*. These data will be published in a forthcoming paper (Michaletz *et al*. in prep).

## Data Records

This paper reports on data from field experiments on fire history and climate impacts on high-elevation Puna grasslands in the eastern Peruvian Andes conducted between 2018 and 2020. It contains data on plant community, vegetation structure, plant functional traits, biomass, ecosystem fluxes, and climate data collected in one or several campaigns between March 2018 and March 2020. Data outputs consist of six datasets, the (i) species composition at the sites along the gradient and from the fire treatments, (ii) vegetation height and structure at the sites along the gradient and from the fire treatments, (iii) biomass harvested in an additional set of plots at five sites in the control and burnt treatment, (iv) plant functional traits of individuals sampled from the sites along the gradient and the fire treatments, (v) ecosystem fluxes from the sites along the gradient and fire treatments and (vi) TOMST logger temperature and soil moisture data from each site and treatment (Table 1). These data were checked and cleaned according to the procedures described under the section Technical validation below before final cleaned data files and associated metadata were produced.

The final cleaned data files (see Table 1 for an overview), data dictionaries, and all raw data, including leaf scans, are available at Open Science Framework (OSF)^70^. To ensure reproducibility and open workflows, the code necessary to access the raw data and produce these cleaned datasets, along with a readme file that explains the various data cleaning steps, issues, and outcomes, are available in an open GitHub repository, with a versioned copy archived in Zenodo^71^. For detailed information about the data cleaning process we refer to the code and the detailed coding, data cleaning, data accuracy comments, and the associated raw and cleaned data and metadata tables. The Usage Notes section in this paper summarises the data accuracy and cleaning procedures, including caveats regarding data quality and our advice on ‘best practice’ data usage.

### Dataset (i): Plant community composition

**Fig. 3:**
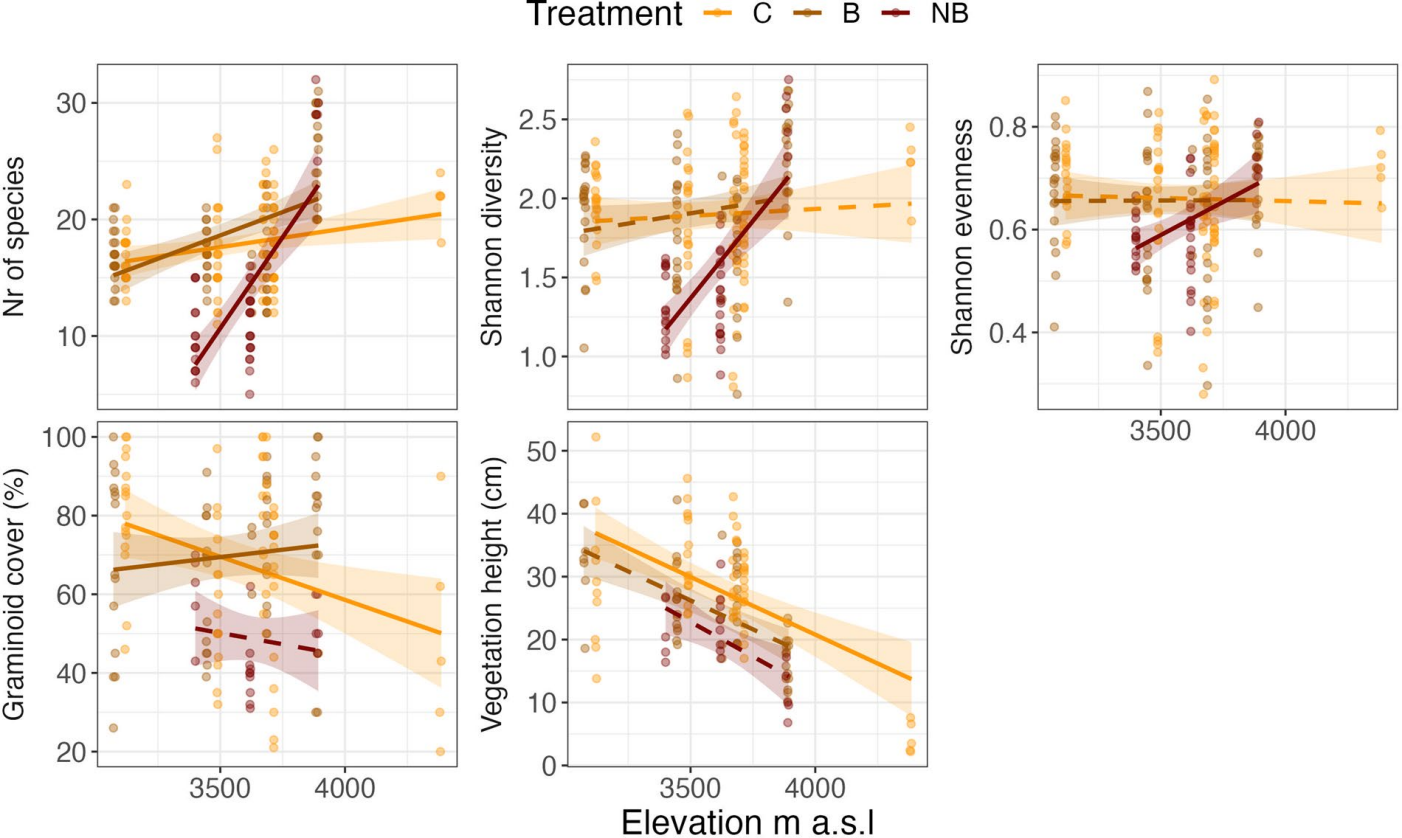
Diversity indices, graminoid cover, and vegetation height from three different fire treatments in six sites along an elevation gradient in the Puna grasslands of Perú. Solid lines indicate a significant relationship with elevation for that fire treatment. Colors indicate the fire treatments: C= control, B = burnt, NB = newly burnt. See text for further explanations.

The plot-level plant community dataset has 145 taxa and 3,665 observations from 63 vegetation plots (taxa x plots x campaign) (Tables 1, 2). Mean species richness per plot and year (mean ± SE) is 17.2 ± 0.37 species, and richness increases by ca. 3 species per 1000 m elevation (E = 0.003, *t*_5,210_ = 2.25, *P* = 0.026). In the burnt plots, richness is lower than controls at low elevations but also increases more towards higher elevations (E = 0.005, *t*_5,210_ = 2.31, *P* = 0.022), and this is especially evident in the recently burnt treatments (E = 0.028, *t*_5,210_ = 6.44, *P* < 0.001; Fig. 3). Diversity and evenness do not change with elevation in the controls, but are lower in the low-elevation recently burnt treatment and also increase with elevation here (diversity: E = 0.002, *t*_5,210_ = 4.50, *P* =  < 0.001; evenness: E = 0.0003, *t*_5,210_ = 2.18, *P* = 0.030; Fig. 3). Graminoid cover is variable and does not change with elevation (E = −0.0003, t_5,210_ = 2.35, P = 0.19); but is generally lower in the newly burnt treatment, where it also decreases with elevation (Fig. 3).

**Table 2:** Data dictionary for the vascular plant community composition (dataset i) from Puna grasslands of the southeastern Andes, in the Manú National Park buffer zone, Department of Cusco, Paucartambo province, Challabamba district, Perú.

Variable name	Description	Variable type	Variable range or levels	Units	How measured
year	Year of sampling	numeric	2018–2020	yyyy	recorded
season	Time of data collection; wet or dry season	categorical	dry_season–wet_season		recorded
month	Month of sampling	categorical	April–November	month	recorded
site	Unique site ID using first three letters of site name	categorical	ACJ–WAY		defined
treatment	Burning treatment; C = control, B = burnt, NB = newly burnt, and BB = experimentally burnt	categorical	B–NB		defined
plot_id	Plot ID	numeric	1–5		defined
family	Plant family name	categorical	Alstroemeriaceae - Violaceae		identified
functional_group	Plant functional group	categorical	Bryophyte - Woody		identified
taxon	Taxon	categorical	Acaena cylindristachya - Zephyranthes sp1		identified
cover	Estimate of individual species cover	numeric	0.5–92	percentage	recorded
burn_year	Year of the latest fire event	numeric	2005–2019	yyyy	recorded
elevation	Elevation of site	numeric	3071.7–4385.8	m asl	recorded
latitude	Latitude of site	numeric	−13.451 −13.12	degree N	recorded
longitude	Longitude of site	numeric	−71.741–71.588	degree E	recorded

The dataset contains 3,665 observations of the covers of 145 taxa in 63 vegetation plots sampled across six sites, three fire histories, and three years. Variable names, description, variable types, range or levels, units, and short descriptions are given for all variables.

For an overview over the cleaned dataset and links to the code to clean and extract these data from the raw data, see Table 1. The final cleaned data can be accessed in the “community” folder, a data dictionary is provided in the “meta” folder, and the raw data can be accessed in the “raw data” folder on OSF^70^. The code to download and clean the data is provided in the GitHub repository^71^ in the file code/2_species_cover.R.

### Dataset (ii): Vegetation height and structure

The dataset on plot-level vegetation height and other structural variables has a total of 1,627 observations (campaign x site x treatment x variable x variable class) (Tables 1, 3, Fig. 3 ). Vegetation height decreases sharply with elevation (E = −0.018, t_5,135_ = −4.80, P < 0.001; Fig. 2), from an average of 31.0 ± 2.41 cm at WAY to 4.46 ± 1.11 cm at OCC but is not affected by the fire treatments. There is also data on the cover of graminoids, ferns, forbs, shrubs, litter, bare ground, and rock, and bryophyte cover and depth, with generally weak responses along elevation and among treatments.

**Table 3:** Data dictionary for the vascular plant community structure variables (dataset ii) from Puna grasslands of the southeastern Andes, in the Manú National Park buffer zone, Department of Cusco, Paucartambo province, Challabamba district, Perú.

Variable name	Description	Variable type	Variable range or levels	Units	How measured
year	Year of sampling	numeric	2018–2020	yyyy	recorded
season	Time of data collection; wet or dry season	categorical	dry_season–wet_season		recorded
month	Month of sampling	categorical	April–March	month	recorded
site	Unique site ID using first three letters of site name	categorical	ACJ–WAY		defined
treatment	Burning treatment; C = control, B = burnt, NB = newly burnt, and BB = experimentally burnt	categorical	B–NB		defined
plot_id	Plot ID	numeric	1–5		defined
burn_year	Year of the latest fire event	numeric	2005–2019	yyyy	recorded
elevation	Elevation of site	numeric	3071.7–4385.8	m asl	recorded
latitude	Latitude of site	numeric	−13.451–13.12	degree N	recorded
longitude	Longitude of site	numeric	−71.741–71.588	degree E	recorded
course	Sampling campaign	categorical	PFTC3–Puna		recorded
variable	Variable name; cover, min, mean and max vegetation height and bryophyte depth	categorical	bryophyte_depth–min_height		defined
variable_class	Variable class; forbs, graminoids, shurb, fern, bryophytes, lichen, bottom-, field-, shrub layer, litter, bare ground and rock (cover), vegetation (height), and bryophyte (depth)	categorical	bare_ground–vegetation		defined
value	Cover, height or depth value	numeric	0–100	percentage or cm	recorded

The dataset reports 1627 observations of the cover of plant functional groups, bare ground and litter, bryophyte layer depth, and vegetation height sampled from 63 plots across six sites, three fire histories, and three years. Variable names, descriptions, variable types, range or levels, units, and short descriptions are given for all variables.

For an overview of the cleaned dataset and links to the code to clean and extract these data from the raw data, see Table 1. The final cleaned data can be accessed in the “community” folder, a data dictionary is provided in the “meta” folder, and the raw data can be accessed in the “raw data” folder on OSF^70^. The code to download and clean the data is provided in the GitHub repository^71^ in the file code/3_community_structure.R.

### Dataset (iii): Plant functional traits

We measured physical and structural traits (plant height, leaf wet mass, leaf dry mass, leaf area, leaf thickness, specific leaf area [SLA], and leaf dry matter content [LDMC]) for 7,609 leaf samples from 121 taxa across all sites and treatments, for a total of 50,264 trait observations (Tables 1, 4). There are variable numbers of leaves per site (WAY = 1,565; ACJ = 2011; PIL = 1,323; TRE = 1,483; QUE = 1,162; OCC = 75) and treatment (C = 3,788; B = 2,641; NB = 1,053 and BB = 137).

**Table 4:** Data dictionary for the plant functional traits (dataset iii) from Puna grasslands of the southeastern Andes, in the Manú National Park buffer zone, Department of Cusco, Paucartambo province, Challabamba district, Perú.

Variable name	Description	Variable type	Variable range or levels	Units	How measured
year	Year of sampling	numeric	2018–2020	yyyy	recorded
season	Time of data collection; wet or dry season	categorical	dry_season–wet_season		recorded
month	Month of sampling	categorical	April–November	month	recorded
site	Unique site ID using first three letters of site name	categorical	ACJ–WAY		defined
treatment	Burning treatment; C = control, B = burnt, NB = newly burnt, and BB = experimentally burnt	categorical	B–NB		defined
plot_id	Plot ID	categorical	1–General		defined
individual_nr	Individual number	numeric	1–10		defined
leaf_nr	Leaf number per individual	numeric	1–7		defined
id	Unique leaf ID	categorical	AAA0656–SVH1234		defined
functionalgroup	Plant functional group	categorical	Fern–Woody		identified
family	Plant family name	categorical	Alstroemeriaceae–Violaceae		identified
taxon	Taxon	categorical	Acaena cylindristachya–Werneria villosa		identified
trait	Plant functional trait	categorical	c_percent–wet_mass_g		defined
value	Leaf trait value	numeric	−34.942–593.892	cm, g, cm^2^, mm, cm^2^/g, percentage, permil	recorded
burn_year	Year of the latest fire event	numeric	2005–2019	yyyy	recorded
elevation	Elevation of site	numeric	3071.7–4384.3	m asl	recorded
latitude	Latitude of site	numeric	−13.451–13.12	degree N	recorded
longitude	Longitude of site	numeric	−71.741–71.588	degree E	recorded
course	Sampling campaign	categorical	PFTC3–Puna		recorded

The dataset contains 54,036 trait observations of 11 structural, economic, and chemical traits from 121 taxa sampled from 63 vegetation plots across six sites, three fire histories, and three years. Variable names, descriptions, variable types, range or levels, units and short descriptions are given for all variables.

Because many specimens had tiny leaves, it was necessary to merge some individuals to obtain enough material for the chemical and nutrient traits (carbon [C], nitrogen [N], phosphorus, C:N and NP ratios, and isotope ratios [d13C, d15N]). A subset of 753 such combined leaf samples from 54 taxa across all sites were thus used for a total of 3,772 chemical or nutrient trait observations. Note that more samples will be added to this dataset as they are processed in the lab.

**Fig. 4:**
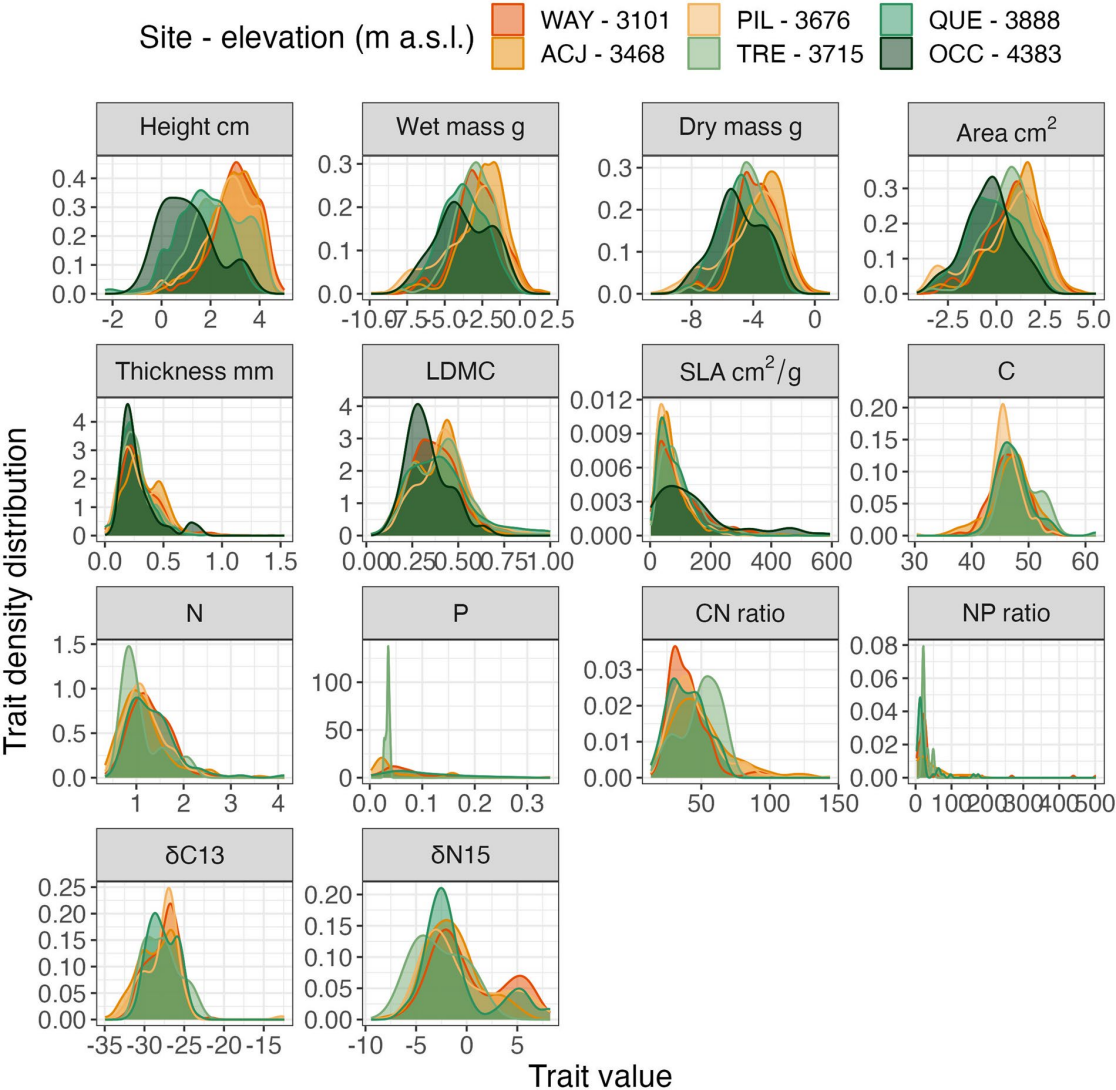
Trait density distributions from six sites along an elevation gradient in the Puna grasslands of Perú. Distributions of trait data (unweighted values) based on all sampled leaves (all fire treatments) per site. The size traits (height, mass, area, and thickness) are log-transformed.

Unweighted trait distributions per site show that “size-related traits” such as height, mass, and area tend to decrease towards higher elevations (Fig. 4). LDMC shows a decreasing trend, indicating more stress-tolerant leaves at higher elevations. SLA does not show a clear trend with elevation.

The dataset is well-suited for exploring weighted trait distributions as we have trait measurements for species making up at least 80% of the cumulative cover for all traits in all plots, following community standards^58^ (calculations based on datasets i). As almost half of the plots (48.2%) meet this criterion for local (plot-level) trait measurements, the data are well suited to explore community-level consequences of intraspecific trait variation.

For an overview over the cleaned dataset and links to the code to clean and extract these data from the raw data, see Table 1. The final cleaned data can be accessed in the “traits” folder, a data dictionary is provided in the “meta” folder, and the raw data can be accessed in the “raw data” folder on OSF^70^. The code to download and clean the data is provided in the GitHub repository^71^ in the file code/1_species_trait_export.R.

### Dataset (iv): Above-ground biomass

The above-ground biomass dataset reports on data from the eight additional plots set up to enable destructive biomass harvest (see above) and has a total of 672 observations (site x treatment x variable x variable class; note that not all treatment-site combinations were sampled; Tables 1, 5). Total plot biomass, and the biomass of forbs, shrubs, bryophyte and litter decrease with elevation whereas graminoids have a non-significant negative trend (total: t_1,38_ = −5.13, *P* < 0.001, forbs: t_1,23_ = −2.17, *P* = 0.043, *P* = 0.070, shrubs: t_1,18_ = −6.39, *P* < 0.001, bryophytes: t_1,33_ = −2.11, *P* = 0.043, litter: t_1,33_ = −2.47, *P* = 0.020, graminoids: t_1,33_ = −1.88).

**Table 5:** Data dictionary for the above-ground biomass (dataset i) from Puna grasslands of the southeastern Andes, in the Manú National Park buffer zone, Department of Cusco, Paucartambo province, Challabamba district, Perú.

Variable name	Description	Variable type	Variable range or levels	Units	How measured
date_of_harvest	Date of biomass harvest	date	2019–04–05–2019–04–13	yyyy-mm-dd	defined
season	Time of data collection; wet or dry season	categorical	wet_season–wet_season		recorded
site	Unique site ID using first three letters of site name	categorical	ACJ–WAY		defined
treatment	Burning treatment; C = control, B = burnt, NB = newly burnt, and BB = experimentally burnt	categorical	B–NB		defined
plot_id	Plot ID	numeric	1–5		defined
burn_year	Year of the latest fire event	numeric	2005–2018	yyyy	recorded
elevation	Elevation of site	numeric	3071.7–3893.1	m asl	recorded
latitude	Latitude of site	numeric	−13.214–13.12	degree N	recorded
longitude	Longitude of site	numeric	−71.641–71.588	degree E	recorded
treatment	Burning treatment: C = control, B = burnt, and NB = newly burnt	categorical	B–NB		defined
variable	Name of variable; biomass, cover, min, mean and max vegetation height and bryophyte depth	categorical	biomass–min_height		defined
variable_class	Name of variable class; forbs, graminoids, shrub, fern, bryophytes, lichen, litter, bare ground and rock (biomass and/or cover), vegetation (height), and bryophyte (depth)	categorical	bae_ground–vegetation		defined
value	Biomass, cover, height or depth value	numeric	0–2273.67	percentage, cm, g	recorded

The dataset contains 129 observations of biomass or cover for different plant groups (graminoids, fern, forbs, shrub, herb, bryophyte, lichen) plus litter, bare ground, and total cover in in eight extra plots sampled in 2019 across five sites and three fire histories. Variable names, descriptions, variable types, ranges or levels, units, and short descriptions are given for all variables.

For an overview of the cleaned dataset and links to the code to clean and extract these data from the raw data, see Table 1. The final cleaned data can be accessed in the “biomass” folder, a data dictionary is provided in the “meta” folder, and the raw data can be accessed in the “raw data” folder on OSF^70^. The code to download and clean the data is provided in the GitHub repository^71^ in the file code/4_biomass.R.

### Dataset (v): Ecosystem fluxes

The dataset on plot-level ecosystem fluxes has a total of 1,673 observations (site x treatment x variable), including 609 CO_2_ and H_2_O flux measurements and 455 soil respiration measurements (Tables 1, 6–8). Across years and seasons, net ecosystem exchange (NEE) varies across sites and ranges from 2.87 ± 0.314 µmols m^−2^ s^−1^ at PIL to 5.07 ± 0.654 µmols m^−2^ s^−1^ at ACJ. In contrast, ecosystem respiration (R_eco_) decreases monotonically towards higher elevations from −3.49 ± 0.261 µmols m^−2^ s^−1^ at WAY to −1.36 ± 0.353 µmols m^−2^ s^−1^ at QUE (no data from OCC). Soil respiration also decreases towards higher elevations, from −1.12 ± 0.0951 µmols m^−2^ s^−1^ at WAY to −4.09 ± 0.786 µmols m^−2^ s^−1^ at OCC. Ecosystem transpiration also decreases towards higher elevations from 2.26 ± 0.130 µmols m^−2^ s^−1^ at PIL to 1.30 ± 0.106 µmols at QUE. These data are raw and not standardised by temperature, PAR and/or biomass. For an overview over the cleaned dataset and links to the code to clean and extract these data from the raw data, see Table 1. The final cleaned data can be accessed in the “flux” folder, a data dictionary is provided in the “meta” folder, and the raw data can be accessed in the “raw data” folder on OSF^70^.

**Table 6:** Data dictionary for the ecosystem _2_ CO_2_ fluxes (dataset v) from Puna grasslands of the southeastern Andes, in the Manú National Park buffer zone, Department of Cusco, Paucartambo province, Challabamba district, Perú. The dataset contains 609 observations of ecosystem CO_2_ fluxes between 2018 and 2020. Variable names, descriptions, variable types, ranges or levels, units and short descriptions are given for all variables.

Variable name	Description	Variable type	Variable range or levels	Units	How measured
year	Year of sampling	numeric	2018–2020	yyyy	recorded
month	Month of sampling	categorical	April– November	month	recorded
day	Day of sampling	numeric	5-27	days	recorded
site	Unique site ID using first three letters of site name	categorical	ACJ–WAY		defined
treatment	Burning treatment; C = control, B = burnt, NB = newly burnt, and BB = experimentally burnt	categorical	B–NB		defined
plot_id	Plot ID	numeric	1–5		defined
flux	Ecosystem carbon flux: NEE = Net ecosystem exchange, Reco = ecosystem respiration, NEE1–3 = net ecosystem exchange during light response curves with 1 being the least and 3 the most shading.	categorical	NEE–Reco		defined
t_start	Start time for model fitting	numeric	1–60	seconds	defined
t_finish	End time for model fitting	numeric	30–100	seconds	defined
c_amb	Average CO_2_ concentration outside the flux tent under ambient conditions measured by the LiCOR	numeric	277.523–457.125	μmols m^−2^ s^−1^	calculated
t_ave	Average temperature inside the flux tent measured by the LiCOR	numeric	−66.254–37.649	Degrees celsius	measured
p_ave	Average pressure inside the flux tent measured by the LiCOR	numeric	64.204–71.141	kilo Pascals	measured
linear_model	CO_2_ flux as slope from linear model of CO_2_ concentration versus time	numeric	−10.926–28.161	μmols m^−2^ s^−1^	calculated
nls_model	CO_2_ flux as slope from non-linear model of CO_2_ concentration versus time	numeric	−49.683–93.991	μmols m^−2^ s^−1^	calculated
linear_rsqd	R square for the linear model	numeric	0–0.998		calculated
nls_sigma	Chi-Squared for the non-linear model	numeric	0.163–4.662		calculated
linear_aic	Akaike Information Criterion (AIC) from the linear model	numeric	−87.266–290.242		calculated
nls_aic	Akaike Information Criterion (AIC) from the non-linear model	numeric	−46.727–418.159		calculated

**Table 7:** Data dictionary for the ecosystem soil respiration (dataset v) from Puna grasslands of the southeastern Andes, in the Manú National Park buffer zone, Department of Cusco, Paucartambo province, Challabamba district, Perú. The dataset contains 455 observations of ecosystem fluxes between 2018 and 2020. Variable names, descriptions, variable types, ranges or levels, units and short descriptions are given for all variables.

Variable name	Description	Variable type	Variable range or levels	Units	How measured
year	Year of sampling	numeric	2018–2020	yyyy	recorded
month	Month of sampling	categorical	April–November	month	recorded
day	Day of sampling	numeric	5–27	days	recorded
site	Unique site ID using first three letters of site name	categorical	ACJ–WAY		defined
treatment	Burning treatment; C = control, B = burnt, NB = newly burnt, and BB = experimentally burnt	categorical	B–NB		defined
plot_id	Plot ID	numeric	1–5		defined
flux	Ecosystem carbon flux: Rsoil = soil respiration	categorical	Rsoil–Rsoil		defined
collar_position	Position of the PVC collar within the plot. A = top right corner, B = bottom left corner	categorical	A–B		defined
t_start	Start time for model fitting	numeric	5–30	seconds	defined
t_finish	End time for model fitting	numeric	40–90	seconds	defined
date	Date of the measurement	categorical	05.04.19– 27.11.19	yyyy-mm-dd	recorded
time	Time of the measurement			hh-mm-ss	recorded
t_start_recording	Start time for measurement	categorical	10:55:01–22:37:13	hh:mm:ss	recorded
t_finish_recording	End time for measurement	categorical	10:56:11 – 22:38:24	hh:mm:ss	recorded
collar_heigth_ave	Average collar height for volume calculation	numeric	0.044–0.092	cm	measured
t_ave	Average temperature inside the flux tent measured by the LiCOR	numeric	39.359–51.399	Degrees celsius	measured
p_ave	Average pressure inside the flux tent measured by the LiCOR	numeric	55.776–70.115	kilo Pascals	measured
w_ave	Average water flux inside the flux tent measured by the LiCOR	numeric	9.623–49.371	μmols m^−2^ s^−1^	calculated
linear_model	Soil respiration as slope from linear model of CO2 concentration versus time	numeric	−12.331–0.188	μmols m^−2^ s^−1^	calculated
linear_model_rsqd	R-squared for the linear model	numeric	0.503–0.999		calculated
linear_model_aic	Akaike Information Criterion (AIC) from the linear model	numeric	−3971.3–455.7		calculated

**Table 8:** Data dictionary for the ecosystem H2O flux (dataset v) from Puna grasslands of the southeastern Andes, in the Manú National Park buffer zone, Department of Cusco, Paucartambo province, Challabamba district, Perú. The dataset contains 609 observations of ecosystem H2O fluxes between 2018 and 2020. Variable names, descriptions, variable types, ranges or levels, units and short descriptions are given for all variables.

Variable name	Description	Variable type	Variable range or levels	Units	How measured
year	Year of sampling	numeric	2018–2020	yyyy	recorded
month	Month of sampling	categorical	April–November	month	recorded
day	Day of sampling	numeric	5–27	days	recorded
site	Unique site ID using first three letters of site name	categorical	ACJ–WAY		defined
treatment	Burning treatment; C = control, B = burnt, NB = newly burnt, and BB = experimentally burnt	categorical	B–NB		defined
plot_id	Plot ID	numeric	1–5		defined
flux	Ecosystem water flux: E = evaporation, ET = evapotranspiration	categorical	E–ET3		defined
t_start	Start time for model fitting	numeric	1–20	seconds	defined
t_finish	End time for model fitting	numeric	40–90	seconds	defined
w_amb	Average water flux outside the flux tent under ambient conditions measured by the LiCOR	numeric	6.878–44.835	μmols m^−2^ s^−1^	calculated
t_ave	Average temperature inside the flux tent measured by the LiCOR	numeric	−66.254–37.649	Degrees celsius	measured
p_ave	Average pressure inside the flux tent measured by the LiCOR	numeric	64.204–71.141	kilo Pascals	measured
c_amb	Average CO_2_ concentration outside the flux tent under ambient conditions measured by the LiCOR	numeric	261.254–444.187	μmols m^−2^ s^−1^	calculated
linear_model	Water flux as slope from linear model of H_2_O concentration versus time	numeric	−1.39–4.286	mmols m^−2^ s^−1^	calculated
nls_model	Water flux as slope from non-linear model of H_2_O concentration versus time	numeric	−7.775–4.814	μmols m^−2^ s^−1^	calculated
linear_rsqd	R square for the linear model	numeric	0.007–0.999		calculated
nls_sigma	Chi-Squared for the non-linear model	numeric	0.014–1.507		calculated
linear_aic	Akaike Information Criterion (AIC) from the linear model	numeric	−427.055–70.057		calculated
nls_aic	Akaike Information Criterion (AIC) from the non-linear model	numeric	−393.554–259.994		calculated

### Dataset (vi): Climate data

The climate dataset contains plot-level air, ground and soil temperature, and soil moisture data corrected for soil temperature between April 2019 and March 2020 (Dataset vi). The full dataset contains 761,624 observations. For details on the cleaned dataset and the code to clean and extract these data from the raw data, see Table 2, 9, which report data summaries for this dataset (see Climate data validation section in Technical Validation).

**Table 9:** Data dictionary for the climate data (dataset vi) from Puna grasslands of the southeastern Andes, in the Manu National Park buffer zone, Department of Cusco, Paucartambo province, Challabamba district, Perú.

Variable name	Description	Variable type	Variable range or levels	Units	How measured
date_time	Date and time of measurement	date_time	2019-04-04 01:15:00 - 2020–03-15 20:45:00	yyyy-mm-dd-hh-mm-ss	recorded
site	Unique site ID using first three letters of site name	categorical	ACJ–WAY		defined
treatment	Burning treatment; C = control, B = burnt, NB = newly burnt, and BB = experimentally burnt	categorical	B–NB		defined
plot_id	Plot ID	numeric	1–5		defined
variable	Microclimate variable	categorical	Air temperature - soil temperature		defined
value	Air, ground, soil temperature or soil moisture per plot	numeric	−10.375–40.5	°C, (m^3^ water × m^−3^ soil) × 100	measured
unit	Variable unit with °C for temperature and (m^3^ water × m^−3^ soil) × 100 for moisture.	categorical			defined
raw_soilmoisture	Raw soil moisture values	numeric	351–3617		measured
burn_year	Year of the latest fire event	numeric	2005–2019	yyyy	recorded
elevation	Elevation of site	numeric	3071.7–3714.7	m asl	recorded
latitude	Latitude of site	numeric	−13.181–13.12	degree N	recorded
longitude	Longitude of site	numeric	−71.641–71.588	degree E	recorded
logger_id	Unique logger ID	numeric	94191301–94191330		defined
treatment	Burning treatment: C = control, B = burnt, and NB = newly burnt	categorical	B–NB		defined

The dataset contains 761,624 observations of climatic data sampled from 26 vegetation plots across six sites, three fire histories, and two years. Variable names, descriptions, variable types, ranges or levels, units, and short descriptions are given for all variables.

During the one year of measurements, mean daily temperature was lowest during the dry season in August. Daily mean air temperature decreased with increasing elevation (11.6 °C in WAY, 9.11 °C in ACJ, 7.81 °C in PIL, and 8.13 °C at TRE), but did not differ among the fire treatments.

For an overview of the cleaned dataset and links to the code to clean and extract these data from the raw data, see Table 1. The final cleaned data can be accessed in the “climate” folder, a data dictionary is provided in the “meta” folder, and the raw data can be accessed in the “raw data” folder on OSF^70^. The code to download and clean the data is provided in the GitHub repository^71^ in the file code/5_climate_data.R.

## Technical Validation

### Taxonomic validation

During the 3-year data collection period, one co-author (LLVB) was responsible for species identification, taxonomic harmonisation between all datasets, and checking problem specimens. In particular, sterile graminoids or young plants can be difficult to identify. Species that could not be identified in the field were given a descriptive name, and a voucher was made and brought back to the University of Cusco to be identified by experts.

The community taxonomy and trait data were checked and corrected against TNRS (see above). A full species list of all identified species across datasets, including their authority, is also available in the OSF repository in the ‘community’ folder. There are in total 25 unidentified taxa (i.e. for which only functional groups, family or genus are identified), 25 in the plant community (dataset i), and 15 in the traits (dataset iii). Note that unknown taxa were harmonised between the datasets so that, for example, “*Genus sp1*” in the trait dataset is the same as “*Genus sp1*” in the trait dataset.

### Community data validation

We checked and corrected missing or unrealistic cover values against the field notes for typing errors. The data-checking code and outcomes for these various procedures is documented in the code on GitHub^71^.

### Trait data validation

This section describes our procedure for trait data checking and validation. Missing or erroneous sample identifications in one or more of the measurements was checked against field notes and notes on the leaf envelopes. Unrealistically high or low values of one or more trait values were checked against the lab and field notes for typing errors, leaf scans were checked for issues arising during the scanning process (e.g., empty scans, double scans, blank areas within the leaf perimeter, dirt or other non-leaf objects on scans). We corrected all issues that could be resolved with certainty (e.g., recalculating leaf area manually for missing leaf parts on the scan, the wrong match between scan and leaf ID, etc.). Any remaining samples with unrealistic trait values that could potentially result from measurement errors were removed (n = 291 values). These include leaves with clearly erroneous leaf area values, leaf dry matter values higher than 1 g/g, specific leaf area values greater than 600 cm^2^/g, carbon content higher than 65%, and negative P content (see the code^71^ for details). Finally, we checked for outliers by plotting the data (e.g., leaf wet mass vs. leaf dry mass). The code for and outcomes of these various procedures are documented and available in the code^71^.

### Climate data validation

The climate data of each plot was inspected, and entries were removed based on quantile ranges of soil moisture, which contained notable jumps indicating the placement of the loggers in the field. Dates at which quantile ranges indicated first placement in the field were extended by an additional day to circumvent measurement errors during placement and allow acclimatisation of the measurement equipment. All data are available, with data before logger placement in the field being marked with error flags.

## Usage Notes

### Data use and best practice

The data are provided under a CC-BY licence. We suggest that data presented here and accessed through the OSF^70^, including future additions to the chemical trait data, be cited to this data paper. We appreciate being contacted for advice or collaboration, if relevant, by users of these data. In cases where our data make up > 10% of the data used in downstream publications we anticipate that appropriately acknowledging our contributions would result in an invitation for collaboration.

### Taxonomic notes

To properly use these data, be aware that the taxonomy of Puna grasslands is challenging because there is no comprehensive identification literature available, and there might be misidentifications in both community and trait data. Note that unidentified taxa are harmonised across datasets.

### Data quality comments and options

This paper and the associated code describe and implement our suggested data management, cleaning, and checking procedures, producing what we consider the clean and ‘best practice’ final datasets from the Puna grassland projects and courses. The various ‘flag’, ‘comment’ and’notes’ columns in the dataset tables (Tables 2–7) give further information about data points that could be used to create more or less restrictive cleaning procedures. Users who prefer stricter or more inclusive data handling strategies should check the flags in the raw data sets and adjust their data cleaning accordingly.

In the traits data, we follow community best practices for ensuring data quality. We filter out what we consider unreliable data points, e.g., leaf dry mass larger than leaf wet mass, leaf areas from erroneous scans, and obviously unrealistic measurements (see above). All cleaning is done using the code available on GitHub, and users are encouraged to check our data cleaning procedures to ensure that the cleaned data fulfil their research needs. Note that the data for some specimens are incomplete (i.e., there may be LDMC or SLA values but no leaf specific mass or area) because of bulk sampling of small leaves. Due to lab costs and bulk sampling of small leaves, chemical data are only available from a subset of leaves.

Due to COVID-19 disruption of the March 2020 traits course^36,37^, species were only partially sampled in the ACJ and WAY sites in that year, focussing on target species for intraspecific trait variability sampling (*Halenia umbellata, Lachemilla orbiculata*, *Paspalum bonplandianum, Rhynchospora macrochaet*a, *Gaultheria glomerata and Vaccinium floribundum*) and some other easily identifiable species (*Lachemilla orbiculata, Eriosorus cheilanthoides, Elaphoglossum huacsaro, Hieracium c.f. mandonii, Baccharis genistelloides, Carex pichinchensis, Elaphoglossum amphioxys, Chaptalia cordata, Miconia rotundifolia, Lycopodium clavatum*). Also, for the same reason, there was no community composition sampling at any sites.

For dataset v, ecosystem fluxes, if users want to set more or less restrictive data exclusion thresholds for fluxes to include in analysis, this can be done from the raw data available on OSF^70^. For example, users might want to visually inspect individual measurements and set different timeframes, use other calculations, depending on their own criterias or research goals.

For dataset vi, the climate data, we used an intermediate soil type “sandy loam A” provided by TOMST to convert raw soil moisture data to the final soil moisture provided in the clean data. Other conversions are possible and can be conducted using the raw soil moisture values in the raw data.

### Spanish language availability

A Spanish language version of this paper is available at https://zenodo.org/records/10581707.

## Data Availability

The code used for checking, cleaning, and analysing the data are available in the open GitHub repository (https://github.com/Plant-Functional-Trait-Course/pftc3_punaproject_pftc5), of which a versioned copy is available at Zenodo^71^. There is also a link to the code from the published dataset^71^.
